# A Rare Cause of Alveolar Echinococcal Metastasis

**DOI:** 10.1590/0037-8682-0435-2022

**Published:** 2023-02-20

**Authors:** Mustafa Yesilyurt, Gökhan Polat

**Affiliations:** 1 Ataturk University, Medical Faculty, Department of Radiology, Erzurum, Turkey.

**FIGURE 1:**
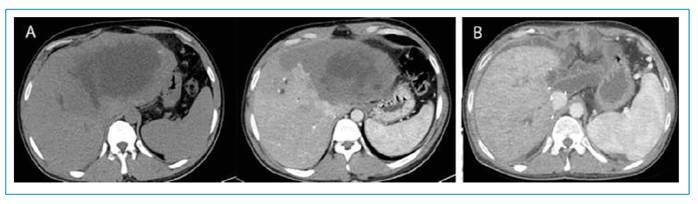
Axial non-contrast and contrast-enhanced computed tomography (CT) images **(A)** show a hypodense mass lesion in the left lobe of the liver before transplantation. Axial contrast-enhanced CT imaging after liver transplantation **(B)**.

**FIGURE 2:**
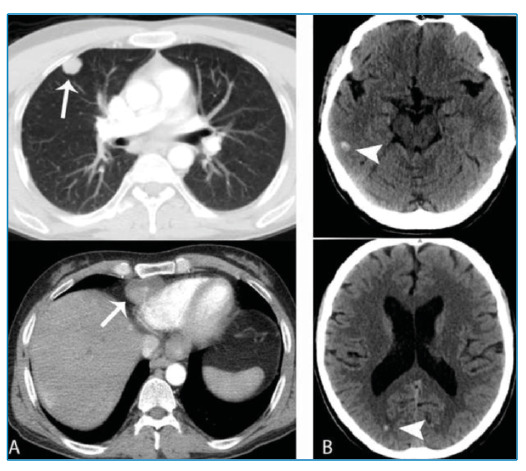
Thorax CT imaging after the transplantation **(A)** shows hypodense mass lesions (arrows) in the paracardiac area and within the lung parenchyma. Axial non-contrast brain CT images after the transplantation **(B)** show hyperdense lesions in the brain (arrowheads).

A 27-year-old male patient was referred to the general surgery clinic of our hospital with a diagnosis of an alveolar echinococcosis cyst. After the examination and imaging, liver transplantation was performed (Figure 1). No echinococcal lesions were found in the other parts of the body before the transplant surgery. After the surgery, the patient received immunosuppressive treatment. Brain computed tomography (CT) and thoracic CT were performed 1 year after transplantation. Multiple solitary lesions were observed in the brain and lung parenchyma (Figure 2). The lesions were biopsied and diagnosed as alveolar echinococcal metastasis.

Alveolar echinococcosis is a chronic and malignant parasitic infection caused by helminth echinococcosis multilocularis^1^. It mostly affects the liver. Radical hepatic resection is the most effective treatment for liver cancers. Liver transplantation can be used as a curative method for the treatment of unresectable cases^1^. However, immunosuppressive treatments used after transplantation may rarely cause metastatic involvement in alveolar echinococcosis^2^. Herein, we report a rare case of alveolar echinococcosis that recurred as lung and brain metastases sometime after liver transplantation. Although resection is the potential treatment that offers the best survival in alveolar echinococcosis, it is revealed that a significant increase in metastasis and spread to surrounding tissues may occur as a result of current immunosuppressive therapy^3^. Therefore, the precise role and duration of perioperative immunosuppressive therapy need to be examined.
